# Nail dystrophy successfully treated with dupilumab in pediatric atopic dermatitis: case series and literature review

**DOI:** 10.3389/fimmu.2026.1668182

**Published:** 2026-02-17

**Authors:** Qian Wang, Jie Liu, Ge Yang, Wei Liu, Xiyuan Zhou, Lixia Zhang

**Affiliations:** 1 Institute of Dermatology and Venereology, Sichuan Provincial People’s Hospital University of Electronic Science and Technology of China, Chengdu, China; 2 Department of Pharmacy, Sichuan Provincial People’s Hospital, School of Medicine, University of Electronic Science and Technology of China, Chengdu, China

**Keywords:** Atopic dermatitis, Dupilumab, Nail changes, Nail dystrophy, Onychodystrophy

## Abstract

Atopic dermatitis (AD) is a common chronic, recurrent inflammatory disease, yet its accompanying nail abnormalities have long received insufficient attention. The clinical characteristics, underlying mechanisms, and assessment systems for nail dystrophy in AD remain unclear. AD-associated nail dystrophy can manifest in various forms, including Beau’s lines, nail pitting, koilongchia, trachyonychia, leukonychia, brachyonychia, melanoychia, onychomadesis, onychoschizia, onycholysis, and paronychia. Current treatments face limitations such as slow onset of action and uncertain efficacy with traditional therapies, particularly with limited drug options for the pediatric populations. With the deepening of research into the Th2 inflammatory pathway, biologics such as dupilumab have shown therapeutic potential. Through retrospective analysis, this paper presents the effectiveness and safety of dupilumab in five pediatric AD patients with nail dystrophy under 12 years old. After at least 12 weeks of treatment, their skin lesions and nail dystrophy both showed marked improvement. Additionally, we reviewed four reported cases in the literature of adult AD patients with nail dystrophy who experienced significant improvement in nail changes after dupilumab treatment. These results suggest that dupilumab may be an effective treatment for nail dystrophy in AD. This case series provides the first evidence demonstrating the significant efficacy of dupilumab for nail dystrophy in pediatric AD patients. However, further large-scale prospective studies are still needed to better guide clinical practice.

## 1 Introduction

As an important window into human health, nails not only serve the aesthetic function of enhancing appearance, but also possess crucial physiological value in protection, tactile feedback, and aiding precision movements. When nails undergo pathological changes, they may render certain daily activities difficult for patients, thereby adversely affecting their quality of life across multiple dimensions—including psychological distress, impaired social functioning, and increased family care burdens. In terms of disease diagnosis, nail abnormalities can serve as early indicators of systemic diseases. Even subtle changes in nail plate morphology, color, or structure can provide important clues for clinical diagnosis. Therefore, nail examination should be an indispensable part of a comprehensive physical examination. However, in clinical practice, this important sign is often overlooked.

Several inflammatory skin diseases can be accompanied by nail changes, such as psoriasis, lichen planus, alopecia areata, and atopic dermatitis (AD). Among these, nail psoriasis has been extensively studied due to its high incidence rate, and a well-established assessment system has been developed. However, as a highly prevalent disease, academic attention to nail changes in AD has lagged behind research on skin lesions and comorbidities. Recent studies indicated that approximately 11% of AD patients exhibited nail manifestations, which might manifest as pitting, melanonychia, chronic paronychia, and hangnail (1). Precise identification of these signs is crucial for disease assessment. The current treatment landscape for AD-related nail disorders presents significant challenges. Traditional therapies have limitations including being slow-acting, ineffective, and difficult to maintain. Particularly in the pediatric population, there is a significant lack of treatment options. As research into the underlying mechanisms of AD deepens, emerging therapies such as biologics and small molecule drugs are offering new prospects for treating nail disorders in AD (2–4). While there have been a few case reports of dupilumab effectively treating nail dystrophy in adult AD patients, there is a lack of data on its efficacy and safety in children.

Here, we for the first time present a retrospective analysis of 5 cases of children under 12 years old with AD and nail dystrophy who experienced satisfactory improvement in both skin lesions and nail symptoms after using dupilumab. Our aim is to describe the manifestations of nail dystrophy in pediatric AD patients and this innovative treatment strategy to provide guidance and reference for clinical practice.

## 2 Case presentation

**Figure 1:**
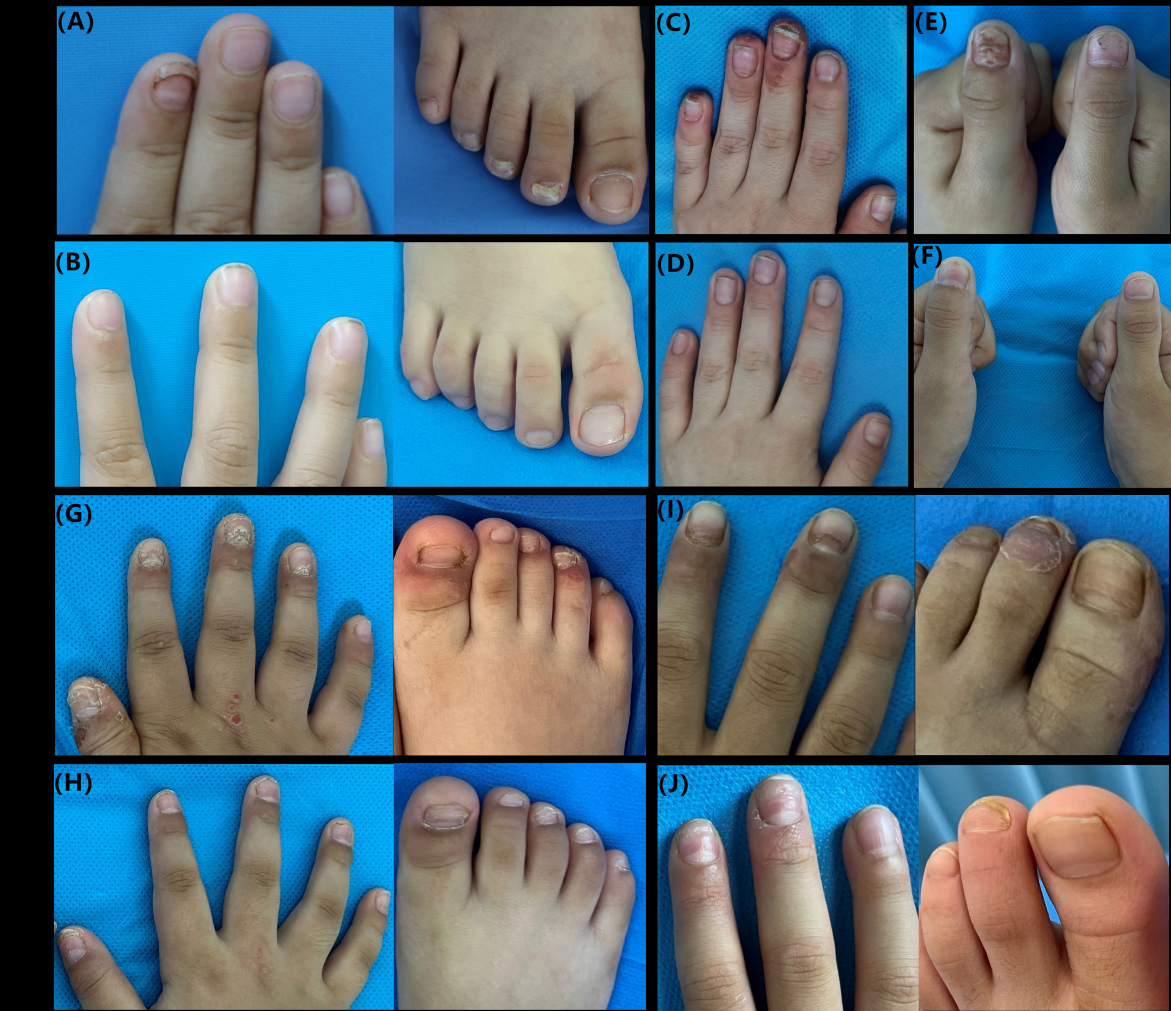
Clinical images of nail manifestations before and after 12 weeks (patient 1, 3, 4, 5) or 24 weeks (patient 2) treatment of dupilumab in 5 pediatric AD patients with nail dystrophy. **(A, B)** Patient 1: Onychomadesis and leukonychia **(A)** almost disappeared after 12 weeks of dupilumab treatment **(B)**. **(C, D)** Patient 2: Beau’s line, chronic paronychia, pitting, hyperkeratosis and erythema of hyponychium, loss of adhesion of the cuticle to the nail plate were seen at baseline **(C)**. After 24 weeks of dupilumab treatment, nail changes had essentially returned to normal **(D)**. **(E, F)** Patient 3: After 12 weeks of dupilumab treatment, transverse ridges along the midline of the left thumbnail plate and onycholysis on the surface of the right thumbnail **(E)** had improved significantly **(F)**. **(G, H)** Patient 4: Her toenail paronychia, trachyonychia and onycholysis of fingernails **(G)** almost resolved after 12 weeks of dupilumab treatment **(H)**, although some toenails still exhibiting transverse grooves and onycholysis **(H)**. **(I, J)** Patient 5: Onychomadesis, Beau’s line and proximal trachyonychia **(I)** partly recovered, with only mild Beau’s line **(J)**.

Patient 1, a 4-year-old girl, presented to our department with “erythematous papules on the limbs accompanied by itching for over six months”. She was diagnosed with eczema locally and treated with moisturizer and topical corticosteroid cream, but her symptoms did not improve significantly. Symptoms worsened after sun exposure. Her father has a history of eczema. Her parents denied her history of other allergic conditions. Physical examination revealed scattered erythematous papules on the limbs, along with onychomadesis and leukonychia affecting fingernails and toenails (**Figure 1A**). Eosinophil (EOS) count was within normal range, while immunoglobulin E (IgE) was 923 IU/ml (normal range: 0–60 IU/ml). Fungal microscopy and culture of the nails were negative. Her parents refused nail biopsy. Based on the patient’s medical history and clinical presentation, a diagnosis of AD with nail dystrophy was made. At the time of presentation, her scores of Scoring Atopic Dermatitis (SCORAD), Eczema Area and Severity Index (EASI), Peak Pruritus Numerical Rating Scale (PP-NRS) and Children’s Dermatology Life Quality Index (CDLQI) were 35.3, 10.4, 6, and 10, respectively. After thorough communication with the parents, treatment with dupilumab (300 mg every 4 weeks) was initiated. After 12 weeks of treatment, she showed significant improvement across multiple AD assessment dimensions (SCORAD 3.7, EASI 1.4, PP-NRS 1, CDLQI 0). Notably, the abnormal nails were largely replaced by healthy nails (**Figure 1B**). During treatment, she developed mild conjunctivitis, which resolved after symptomatic management. No other adverse events were observed. At the 30-week follow-up, the therapeutic effect remained stable.

Patient 2, a 6-year-old boy with a 3-year history of AD had previously been treated with oral cetirizine and topical corticosteroid creams but without success. Both the patient and his parents had allergic rhinitis. Physical examination revealed scattered erythema, papules, nodules, and hyperpigmentation on the trunk, limbs, hands, and feet, with some areas showing erosion and crusting. Additionally, Beau’s line, chronic paronychia, pitting, hyperkeratosis and erythema of hyponychium, and loss of adhesion of the cuticle to the nail plate were observed on his nails (**Figure 1C**). EOS count was normal, and IgE was elevated at 708 IU/ml (normal range: 0–60 IU/ml). Fungal microscopy and culture of the nails were negative. His parents declined nail biopsy. He was diagnosed with AD with nail dystrophy based on his clinical features. With parental consent, the patient was treated with dupilumab (600 mg initial dose, then 300 mg every 4 weeks). Baseline assessment scores were: SCORAD 41.3, EASI 16.1, PP-NRS 7, and CDLQI 10. At the 24-week follow-up, the patient exhibited significant improvement in lesions and pruritus (SCORAD 9.8, EASI 1.7, PP-NRS 2, CDLQI 2), and the nail changes had essentially returned to normal (**Figure 1D**). No adverse effects of dupilumab were noted during treatment.

Patient 3, a 7-year-old boy with a 4-year history of AD presented with recurrent flares. Previous treatments with oral loratadine and topical corticosteroids provided partial relief, but the condition recurred repeatedly. Symptoms worsened with milk consumption and sweating. His personal history included allergic rhinitis and asthma, and his father had a history of allergic rhinitis. Physical examination revealed widespread erythema, papules, plaques, erosion, and crusting over the body, as well as multiple transverse ridges along the midline of the left thumbnail plate and onycholysis on the surface of the right thumbnail (**Figure 1E**). Laboratory investigation showed EOS 0.915×10^9^/L (normal range: 0.00-0.68×10^9^/L) and IgE 701 IU/ml (normal range: 0–60 IU/ml). Fungal microscopy and culture of the nails were negative. His parents refused nail biopsy. According to his medical history and clinical manifestation, a diagnosis of AD with nail dystrophy was confirmed. At baseline, his score of SCORAD was 67.6, EASI was 31.8, PP-NRS was 8 and CDLQI was 30. Treatment with dupilumab (600 mg initial dose, then 300 mg every 4 weeks) was initiated with parental consent. After 12 weeks of treatment, the patient showed improvement in all assessment scores (SCORAD 44.4, EASI 12.2, PP-NRS 4, and CDLQI 10), and the affected nails had almost completely returned to normal (**Figure 1F**). During treatment, the patient developed mild conjunctivitis, which improved after symptomatic management, and dupilumab therapy was not discontinued.

Patient 4, a 4-year-old girl, presented with generalized erythematous papules and nail damage for 3 years. Treatment with oral cetirizine and topical corticosteroids failed to adequately control the condition. Consumption of milk, eggs, and wheat exacerbated the symptoms. Her parents denied her history of other allergic conditions. Her mother and her sister have a history of eczema. EOS and IgE levels were within normal ranges. Physical examination revealed generalized erythema, papules, plaques, erosion, and crusting on the trunk and limbs. Nails exhibited trachyonychia, onycholysis, hyponychium hyperkeratosis, and chronic toenail paronychia (**Figure 1G**). Fungal examinations (direct microscopy and culture) of the nails were negative. Her parents refused nail biopsy. Based on her medical history and clinical presentation, the diagnosis was AD with nail dystrophy. After her parents’ consent, dupilumab (300 mg every 4 weeks) was given. At baseline, her scores of SCORAD, EASI, PP-NRS, CDLQI were 55.2, 21.4, 8, 24, respectively. After 12 weeks of treatment, the patient showed significant clinical improvement achieving the scores of SCORAD, EASI, PP-NRS, CDLQI to 21.2, 4.6, 4, 6, respectively. Apart from some toenails still exhibiting transverse grooves and onycholysis, her paronychia almost resolved, and the previously affected fingernails grew out distally, with gradually normalization of the nail appearance (**Figure 1H**). No significant adverse reactions were observed during treatment. At the 24-week follow-up, the condition of the nails and skin lesions remained well-controlled.

Patient 5, an 8-year-old boy, had a 1-year history of generalized erythematous papules and nail damage, with poor response to conventional antihistamines and topical corticosteroids. Both the patient and his brother had a history of allergic rhinitis. EOS and IgE levels were within normal ranges. Physical examination revealed scattered erythema and papules with erosions and crusts over the body. Onychomadesis, proximal trachyonychia were observed on her fingernail plates and Beau’s line on her toenails (**Figure 1I**). Fungal microscopy and culture of the nails were negative. His parents refused nail biopsy. According to the clinical features and history, the diagnosis was AD with nail dystrophy. After 12 weeks of treatment with dupilumab (600 mg initial dose, then 300 mg every 4 weeks), the patient demonstrated marked clinical improvement (SCORAD 29 vs. 9.4, EASI 6.8 vs. 2.4, PP-NRS 6 vs. 2, CDLQI 5 vs. 2). At the same time, the affected nails partly recovered, with only mild Beau’s line and pitting (**Figure 1J**). No significant adverse reactions were observed during treatment.

**Figure 2:**
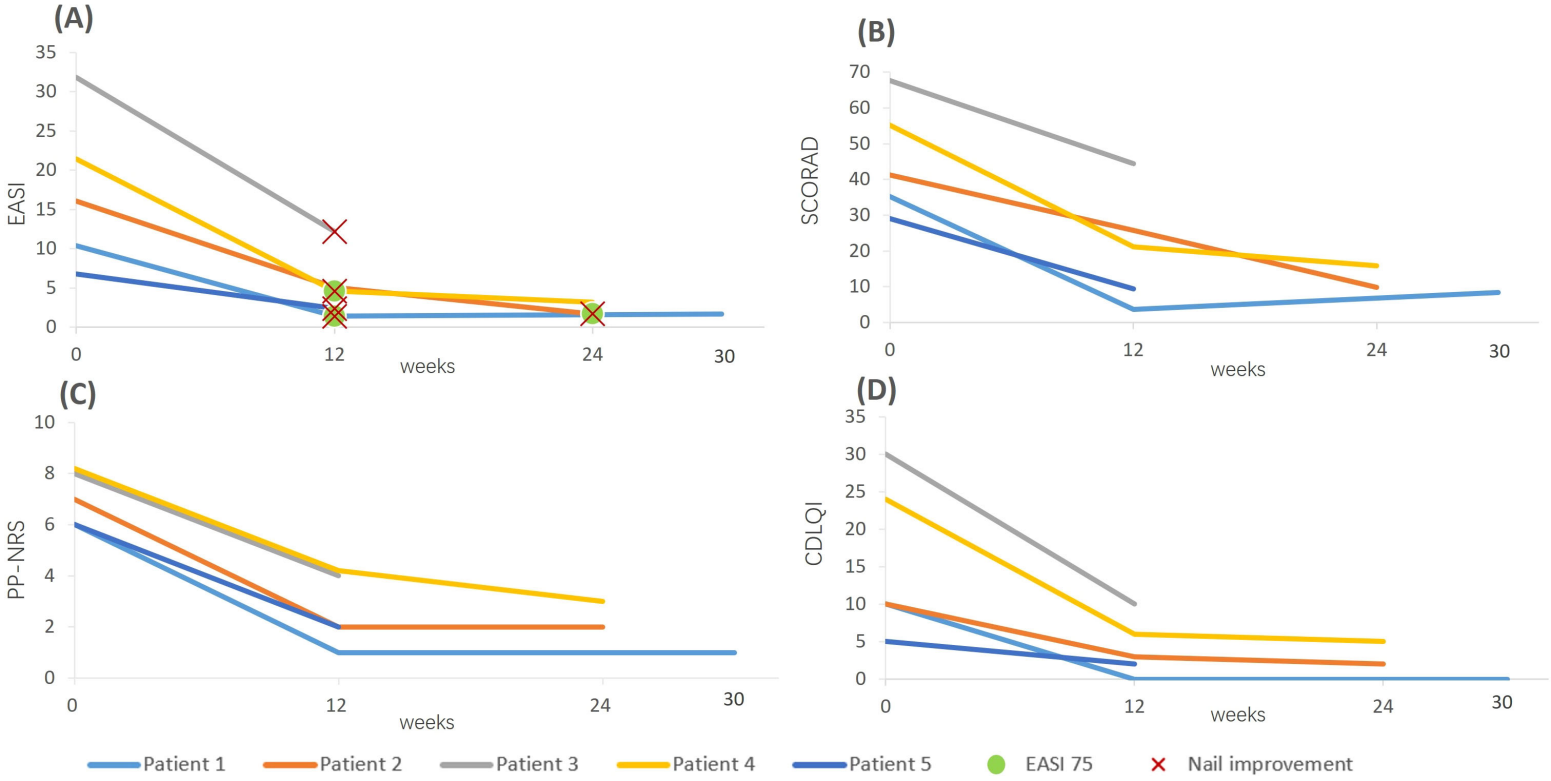
Changes in AD-related clinical scores of the five pediatric patients at different follow-up time points after dupilumab treatment. **(A)** Follow-up time points for EASI scores and obvious improvement in nail dystrophy: Patients 1 and 4 achieved EASI 75 at the 12-week follow-up; patient 2 achieved it at the 24-week follow-up. The first follow-up time point showing significant improvement in nail dystrophy was 12-week for patients 1, 3, 4, and 5, and 24-week for patient 2. **(B–D)** Changes in SCORAD **(B)**, PP-NRS **(C)**, and CDLQI **(D)** scores at different follow-up time points.

The clinical characteristics of the five patients are summarized in **Table 1** and **Figure 2** as follows.

**Table 1:** Clinical characteristics of five pediatric AD patients with nail dystrophy treated with dupilumab in this study.

Patient	1	2	3	4	5
Age(years)	4	6	7	4	8
Gender	Female	Male	Male	Female	Male
AD duration (years)	0.5	3	4	3	1
Allergic comorbidities	/	allergic rhinitis	allergic rhinitis and asthma	/	allergic rhinitis
Familiar background of atopic diseases	Father: eczema	Parents: allergic rhinitis	Father: allergic rhinitis	Mother and sister: eczema	Brother: allergic rhinitis
Nail manifestation	Onychomadesis, leukonychia	Beau’s line, chronic paronychia, pitting, hyperkeratosis and erythema of hyponychium, loss of adhesion of the cuticle to the nail plate	multiple transverse ridges along the midline of the left thumbnail plate and onycholysis on the surface of the right thumbnail	trachyonychia, onycholysis, hyponychium hyperkeratosis, and chronic toenail paronychia	Onychomadesis, proximal trachyonychia and Beau’s line
Previous treatment	Moisturizer, topical corticosteroids	Cetirizine, topical corticosteroids	Loratadine, topical corticosteroids	Cetirizine, topical corticosteroids	Antihistamine, topical corticosteroids
Dupilumab treatment duration	30 weeks	24 weeks	12 weeks	24 weeks	12 weeks
Setting of administration	Local clinic	Local clinic	Local clinic	Local clinic	Local clinic
Adverse effects	Mild conjunctivitis	/	Mild conjunctivitis	/	/
Outcome of nail abnormalities	Improved	Improved	Improved	Improved	Improved

## 3 Literature review

Four articles on 4 cases of dupilumab treatment in AD patients with nail dystrophy were identified and analyzed using the Google Scholar, PubMed and Web of Science Core Collection databases (conducted up to June 30, 2025) (5–8), see **Table 2**. Specific retrieval terms included: (dupilumab) or (IL-4Rα) or (IL-4/IL-13) AND “atopic dermatitis” AND “nail dystrophy” or “onychodystrophy” or “nail changes”. The cohort comprised 3 females and 1 male, aged between 39 and 60s years. Three patients had previously shown inadequate response to conventional therapies such as immunosuppressants, systemic corticosteroids, and/or topical corticosteroids. Follow-up after dupilumab initiation ranged from 3 to 15 months. All patients demonstrated significant improvement not only in their skin lesions but also in nail dystrophy. Notably, one patient developed new, severe nail changes 3 months after starting dupilumab therapy. However, with continued treatment, the nail symptoms gradually improved (7). To date, no other previous reports specifically addressing dupilumab treatment for nail dystrophy in pediatric AD patients have been identified, except for one case involving a 5-year-old boy whose trachyonychia incidentally improved during dupilumab treatment for AD and alopecia universalis (9). However, given that his AD was controlled and alopecia areata (AA) was progressive, the authors attributed his nail changes to AA rather than AD.

**Table 2:** Clinical features of AD patients with nail dystrophy treated with dupilumab in literature review.

Reference/year	Age (years)	Gender	Atopic disease	Nail changes	Previous treatment	Follow-up time	Outcome of nail abnormalities
Giura MT/2020 (5)	55	Female	Atopic dermatitis	Median canaliform nail dystrophy of Heller	Topical and systemic steroids or ciclosporin	4 months	Improved
Navarro-Triviño FJ/2021 (6)	61	Male	Atopic dermatitis	Onychodystrophy	Methotrexate, cyclosporin, alitretinoin, etanercept and adalimumab	3 months	Improved
Zubek AE/2020 (7)	60s	Female	Atopic dermatitis	Onychodystrophy	No data	15 months	New severe nail changes appeared after 3 months of dupilumab use, but as dupilumab continued to be used, the nail changes gradually improved
Li J/2025 (8)	40	Female	Atopic dermatitis and allergic rhinitis	Twenty-nail dystrophy	Antihistamine, topical halometasone cream, pimecrolimus ointment and oral corticosteroid treatment	5 months	Improved

## 4 Discussion

Atopic dermatitis (AD) is a chronic inflammatory disease characterized by impaired skin barrier function and Th2-dominant immune dysregulation. Clinically, it primarily manifests as recurrent eczematous skin lesions, severe itching, and dry skin. Chronic scratching can lead to lichenification, and lesions are often symmetrically distributed (10). The distribution of affected areas varies with age: in infants and young children, AD commonly affects the face, scalp, and extensor surfaces, while in adults, flexural areas, hands and feet are predominantly involved. Nail changes may occur as a minor feature of AD in some patients (11). Currently, physicians generally pay little attention to nails, and nail changes are often overlooked. There is limited high-quality data on nail disorders in AD, and even less data on treatment.

The reported incidence of nail changes in AD varies. Compared to patients with psoriasis, nail abnormalities are less common in AD. Up to 50% of psoriasis patients experience nail involvement, with a lifetime incidence of nail psoriasis approaching 90% (12). Chng et al. recently published a review to explore the prevalence of nail disorders seen in AD. Through a systematic review of 7575 cases of AD from 12 studies, it was found that the prevalence of nail disease in AD patients was 11% (1). When the meta-analysis was stratified according to the regions studied (Asian vs. non-Asian studies), the pooled prevalence of nail changes among non-Asian studies was twice that among Asian studies (18.0% vs. 9.0%), suggesting that genetic background, environmental exposure, or cultural habits might affect epidemiological data (1). There are also studies suggesting that the sites of eczema involvement may be associated with the occurrence of nail dystrophy. Simpson et al. found a significant association between AD and chronic hand eczema, with approximately 58.9% of active AD patients experiencing hand involvement and 16% experiencing nail dystrophy (13). Another study showed a prevalence of nail dystrophy of 32.3% in patients with hand eczema, and the disease duration was significantly longer in patients with nail changes compared to those without (14). Some scholars have suggested that the periungual eczema and twenty-nail dystrophy (TND) may represent a distinct phenotype of AD. Thus, nail involvement may not be uncommon in AD patients, especially those with hand eczema, and deserve more attention.

Currently, research on the gender and age predilection of nail changes in AD is limited. Some studies indicated nail abnormalities were more common in males than in females (15), while other studies suggested no significant gender difference (16). In terms of age, nail changes may be more common in adult AD patients than in children (17). Studies on hand eczema showed that nail dystrophy predominantly affected individuals in their 30s and 50s (13). The study conducted by Chung et al. on AD patients aged 2–19 years showed that the average age was higher in patients with nail abnormalities than in those without (15). However, a study indicated that nail involvement is more common in children aged 2 to 7 years than in those aged 7 up to 12 years (16).

Diagnosis of nail dystrophy in AD relies primarily on clinical presentation, as nail biopsy carries the possibility of permanent nail dystrophy. So, nail biopsy was rarely performed to confirm histological features of nail apparatus, especially in pediatric patients. Parents of all 5 pediatric AD cases in our study refused biopsy. Nail changes in AD should be differentiated from nail damage caused by trauma, infection, and other inflammatory conditions such as psoriatic nails. AD affects the various aspects of the nail unit via periungual inflammation and surrounding skin changes. The clinical manifestations can provide some clues about the specific area of the nail unit affected. Eczematous changes infiltrating the nail matrix can lead to anonychia, nail pitting, trachyonychia, longitudinal ridges, Beau’s line, onychomadesis, and onychoschizia (18). Damage to the nail bed can result in brachyonychia, chromonychia, koilonychia, onycholysis, and splinter hemorrhage (19). Nail changes in AD may indicate the severity of AD to some extent, but existing research findings lack consistency. Some evidence supported an association between pitting and trachyonychia with higher SCORAD scores (20), and patients with total nail abnormalities had higher average EASI scores than those without (15). However, other studies suggested that the severity of hand eczema did not parallel to the extent of nail changes (14). These inconsistencies may stem from the high heterogeneity of AD.

The pathogenic mechanisms of AD-associated nail dystrophy may include: 1. Acute and chronic nail dystrophy caused by periungual tissue inflammation, 2. Chronic trauma from habitual cuticle manipulation due to chronic itching-scratching (21), 3. Increased exposure to topical irritants such as soap and detergent during treatment (1). These potential mechanisms may collectively form an “inflammation-behavior-environment” pattern. This suggests that, alongside controlling systemic inflammation, enhancing periungual skin care and behavioral interventions are also essential foundations for treating AD-related nail dystrophy.

The traditional treatments for nail dystrophy include direct injection or topical application of corticosteroids (22), topical tacrolimus (23), PUVA phototherapy (24), laser therapy (25), etc., but large-scale clinical trials for these treatments are currently lacking. In recent years, biological agents targeting the type 2 inflammatory pathways have been attempted for treating nail dystrophy in AD, but clinical experience remains insufficient. Dupilumab, an interleukin (IL) -4 receptor subunit α (IL-4Rα) antagonist, inhibits IL-4 and IL-13 signaling, thereby suppressing the Th2 pathway. The published research findings on dupilumab mainly focus on its improvement in AD skin lesions. In the clinical trials involving thousands of patients treated with dupilumab, no reports of any associated nail changes have been documented (7). To date, only four case reports describe the use of dupilumab in adults with coexisting AD and nail dystrophy, all showing favorable efficacy (5–8). There are no relevant reports on pediatric patients. The mechanism by which dupilumab treats nail dystrophy in AD may be due to blocking the inflammatory infiltration formed by Th2 lymphocytes in the nail matrix or nail bed. Notably, in one reported case, dupilumab played a dual role in both initiating and treating the nail dystrophy (7). This paradoxical response might be attributed to dupilumab temporarily altering the local cytokine environment of the nail, triggering the lesions, though inflammation ultimately relieved over time. Therefore, based on the limited available literature, dupilumab appears to be a potential therapeutic option for atopic nail dystrophy. Compared to small molecule drugs, dupilumab offers a broader applicable age range (6 months and above), carries no black box warning, has a favorable safety profile, and does not require specific laboratory monitoring during treatment (26). This makes it a favorable treatment choice for younger children.

In conclusion, nail dystrophy in patients with AD is an underestimated issue. Novel therapies targeting the pathophysiological mechanisms of AD hold great promise for patients. Our case series is the first to demonstrate the remarkable efficacy of dupilumab in treating nail dystrophy associated with AD in children. After at least 12 weeks of treatment, all five pediatric patients showed significant improvement in their nail abnormalities. However, the existing evidence has inevitable limitations: small sample size, lack of histological support and long-term follow-up data, an unclear association between nail dystrophy and the severity or subtype of AD. Additionally, there is currently a lack of quantifiable assessment tools for evaluating nail improvement in AD. Furthermore, several key clinical questions remain unanswered, including the response after treatment discontinuation, the sustainability of nail improvement, and whether its disease course parallels changes in skin lesions. Therefore, future larger-scale, long-term prospective studies are needed to elucidate the molecular mechanisms, establish evaluation standards, and objectively evaluate the efficacy and long-term impact of targeted therapies on nail dystrophy in AD.

## Data Availability

The original contributions presented in the study are included in the article/supplementary material. Further inquiries can be directed to the corresponding authors.
